# Molecular dynamics simulation of aluminum nitride deposition: temperature and N : Al ratio effects

**DOI:** 10.1098/rsos.180629

**Published:** 2018-08-15

**Authors:** Libin Zhang, Han Yan, Guo Zhu, Sheng Liu, Zhiyin Gan

**Affiliations:** 1 School of Mechanical Science & Engineering, Huazhong University of Science & Technology, Wuhan 430074, People's Republic of China; 2 School of Mechanical & Electronic Engineering, Wuhan University of Technology, Wuhan 430070, People's Republic of China; 3 School of Power and Mechanical Engineering, Wuhan University, Wuhan 430072, People's Republic of China

**Keywords:** AlN deposition, temperature, N : Al flux ratio, crystallinity, stoichiometry, stress

## Abstract

Heteroepitaxial growth of aluminum nitride (AIN) has been explored by experiments, but the corresponding growth mechanism is still unrevealed. Here, we use molecular dynamics simulations to study effects of temperature and N : Al flux ratio on deposited AlN. When the temperature increases from 1000 K to 2000 K with an N : Al flux ratio of 2.0, the growth rate of the AlN film decreases. The crystallinity of the deposited AlN is distinctly improved as the temperature increases from 1000 K to 1800 K and it becomes saturated between 1800 K and 2000 K. The crystallinity of the deposited film at 1800 K increases with an increase in the N : Al flux ratio from 0.8 to 2.4, and this degraded a little at an N : Al flux ratio of 2.8. In addition, stoichiometry is closely related to crystallinity of deposited films. Film with good crystallinity is connected with a near 50% N fraction. Furthermore, the average mean biaxial stress and mean normal stress at 1800 K with N : Al flux ratios of 2.0, 2.4 and 2.8 are calculated, indicating that the deposited film with lowest stress has the best crystal quality and the defects appear where stresses occur.

## 1. Introduction

Bestowed with properties of wide band gap (6.2 eV), high thermal conductivity (3.3 WK^−1^ cm^−1^), high electrical resistivity (10^13^ Ωcm) and piezoelectric effect and high strength, AlN is widely used in the area of photoelectric devices, piezoelectric devices, electronic packaging, microelectronic devices and hard coating [1–5]; especially in the field of photoelectric devices, it is extensively used for fabrication of the deep-ultraviolet light-emitting diodes (UV-LEDs) [6–8].

However, because of the high cost of AlN substrate and its limited performance, growing AlN films on a foreign substrate is still the main method. Sapphire [9], SiC [10] and Si [11] are the cardinal substrates. Nevertheless, the lattice mismatch with those materials cannot be ignored, which results in high threading dislocation density, and it is still difficult to reduce threading dislocation density to 10^5^ cm^−2^ [12–16]; therefore it is hard to improve the performance of devices [17–19]. Growing AlN films on AlN substrate with zero lattice mismatch is beyond doubt the best way; however, commercially available AlN substrate is still immature. Thus, the technique for inducing homoepitaxial AlN growth needs to be further exploited.

Moreover, the growth mechanisms concerning homoepitaxial growth of c-plane AlN are still not enough. A lot of scholars only studied homoepitaxial growth of c-plane AlN from experimental aspects, and there is still a long way to go, although some progress has been made [20–22].

Although c-plane is a critical plane for growing AlN [23], there are a few studies concerning the corresponding mechanism of polar c-plane AlN. Similar theoretical studies have been reported on the deposition of GaN [24] and non-polar AlN [25]. Atomic-scale simulation of the growth and its dependency on various deposition conditions will facilitate effective understanding of the c-plane AlN growth process. The atomic assembly mechanism, crystallinity and defects can be visualized directly by molecular dynamics (MD) simulations instead of experiments.

In this paper, the growth of AlN on c-plane AlN is studied by the large-scale atomic/molecular massively parallel simulator [26] with the Tersoff potential [27]. At an N : Al flux ratio of 2.0, the growth temperature is varied from 1000 K to 2000 K with an increment of 200 K. Then the N : Al flux ratio is varied from 0.8 to 2.8 with an increment of 0.4 at 1800 K. The growth rate, crystallinity and stoichiometry under different conditions are discussed in detail. In addition, the reaction mechanism between substrate atoms and adatoms is revealed. Furthermore, the stresses of deposited AlN films are investigated.

## 2. Model and methods

### 2.1. Model and potential

**Figure 1.**
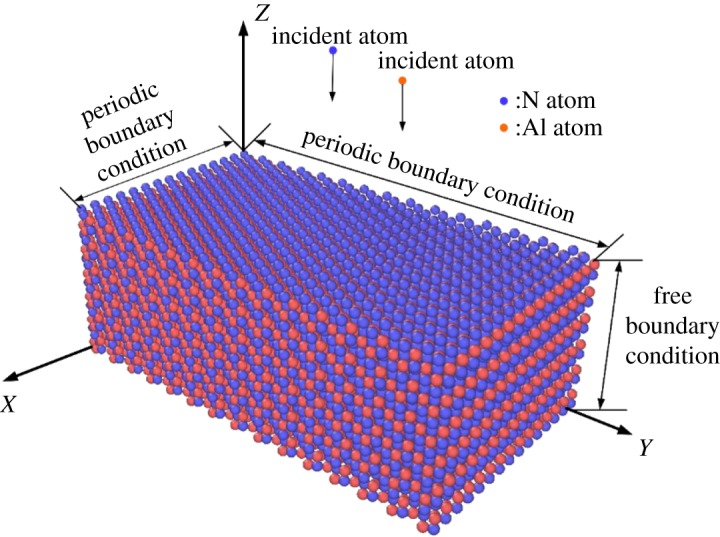
Model of the substrate; the red atoms represent the Al atoms, the blue atoms represent the N atoms.

The three-dimensional model of the AlN substrate is built, as shown in figure 1. *x*, *y* and *z* represent the direction of $<1\bar{1}00>$, $<11\bar{2}0>$ and <0001>, correspondingly. The substrate filled with 10 800 atoms (5400 Al atoms and 5400 N atoms) has a dimension of 46.65 Å, 80.8002 Å and 29.868 Å, accordingly. The red atoms represent the Al atoms, while the blue atoms represent the N atoms. The atoms on the top layers are N atoms. The substrate is divided into three groups: two pairs of closely spaced Al, and N planes along the *z* direction at the bottom are fixed to prevent the moving of the substrate due to the hitting of Al and N atoms during the deposition, which is called the fix group. The middle eight pairs of atoms make up the thermal control group, where the canonical (NVT) ensemble is used to perform time integration on Nose–Hoover style non-Hamiltonian equations of motion to update the positions and velocities of the atoms to get the prescribed substrate temperature [25]. The uppermost two pairs of atoms form the free group in which the atoms are entirely free to interact and transmit energy with the deposited atoms. Periodic boundary conditions are applied to the direction of *x* and *y* in order to create a seemingly infinite boundary in these dimensions, while the free boundary condition is applied to the *z* direction to enable the deposition of Al and N atoms towards the substrate surface. The growth of AlN is simulated by periodically injecting an Al atom or a N atom from a 25-fold-lattice height towards the substrate surface, and the *x* and *y* coordinates of injected atoms are allocated randomly. The incident angle was 0° (perpendicular to the substrate) [28], which is indicated by the black arrow in figure 1. The injected atoms were all assigned a kinetic energy of 0.17 eV. Such thermalized fluxes are analogous to those of a molecular beam epitaxy or a high pressure sputtering process. The three-body Tersoff potential is applied to describe the atomic interactions between atoms, and its reliability and application have already been proved [25,27,29].

The time interval of injected Al atoms is kept as a constant of 5 ps per atom, while that of N atoms is varied to get a range of N : Al flux ratios, ranging from 0.8 to 2.8. The total number of injected Al atoms is 4000, while the injected N atoms are varied according to the N : Al ratio in each simulation. The deposition process takes 20 000 ps. After the deposition, a relaxation process of 10 000 ps is taken to equilibrate the system. The visualization of the model is realized by OVITO [30].

### 2.2. Calculation and analysis methods

The stress of the substrate and deposited films is calculated. Because stress is closely correlated to crystal quality, atom stress can be described by Basinski *et al.* as follows [31]:

2.1 $$\sigma =\frac{1}{V_{i}}\left[m_{i}v_{i}+\frac{1}{2}\sum_{i\neq j}^{N}r_{ij}⊗f_{ij}\right]$$

and

2.2 $$\;\sigma_{mn}^{\mathrm{avg}}=\frac{1}{N}\sum_{i=1}^{N}\sigma_{mn}^{i}.$$

In formula (2.1), *V_i_* represents the volume of the atoms, *m_i_* represents the mass of atom *i, v_i_* represents the velocity of atom *i*, *r_ij_* denotes the distances between atom *i* and the atoms around it, *f_ij_* denotes the inter-atomic forces between them, and ⊗ represents the tensor product between the two vectors.

In formula (2.2), $\sigma_{mn}^{i}$ represents the stress of the *i*th atom, *m* and *n* are indices of the stress tensor, and *N* is the number of atoms in the substrate or the deposited film. Here we calculate the average normal stress $\sigma_{zz}^{\mathrm{avg}}$ and the average mean biaxial stress: $(\sigma_{xx}^{\mathrm{avg}}+\sigma_{yy}^{\mathrm{avg}})/2$. They are selected because the first one is one order of magnitude higher than the shear stress in the deposition case, and the latter is related to the residual stress of the subject, which are interrelated to their structure composition [31,32].

To quantitatively compare the atomic structure of deposited films, dislocation analysis (DXA) [32] is adopted, in which the local environment of each atom is analysed to identify atoms that form a perfect crystal lattice. Atomic structure identification is based on the common neighbour analysis method, which can identify the hexagonal diamond, cubic diamond and other structures. The DXA ignores the chemical atom types, thus the hexagonal diamond (cubic diamond) structure could be treated as Wrutzite (Zinc Blende).

## 3. Results and discussion

### 3.1. Effect of temperature

**Figure 2.**
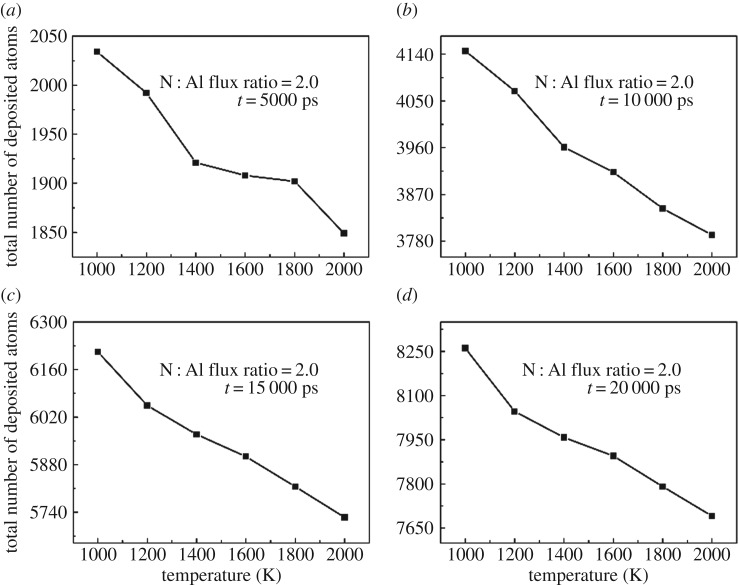
Total number of atoms during and after deposition under different deposition temperatures.

Temperature is a critical factor for growth of AlN, and high temperature is favourable for growing AlN [33]. However, it is still a great challenge to improve the temperature of the reactor chamber higher than 2000 K experimentally. Hence the temperature is changed from 1000 K to 2000 K in order to discuss the effect of growth temperature, and the N : Al flux ratio is set to be 2.0. The growth results under different temperatures are shown in figure 2. The deposition process is divided into four stages by time and the total number of deposited atoms at all stages are calculated as shown in figure 2*a*–*d*. We find that as the temperature increases from 1000 K to 2000 K, the total number of finally deposited atoms decreases. For a certain stage, the total number of deposited atoms decreases as the temperature increases despite the fact that the injected atoms are all the same. The number of deposited atoms indicates the growth rate of AlN films. It is obvious that the growth rate decreases at all stages as the temperature increases from 1000 K to 2000 K. This is owing to the improved desorption effect when the temperature increases [34]. During the deposition, some of the injected atoms adsorb on the surface, while other atoms desorb from the surface owing to thermal fluctuation. The injected Al atoms are mainly consumed to form AlN, while the injected N atoms are easier to desorb when the temperature increases. Increasing the temperature increases the probability of desorption, thus reducing the growth rate.

Crystal lattice perfection is essential for the AlN film. Deviation from the ideal lattice configuration can change the coordinates of atoms and overstretch bonds, which result in changed electron populations in the valence and conduction bands. In addition, the existence of defects and even polytypism seriously affects the electrical and optical properties of AlN [35]. Thus, the perfect lattice, a stoichiometric composition and low defect concentration are expected to produce a high-quality AlN film.

**Figure 3.**
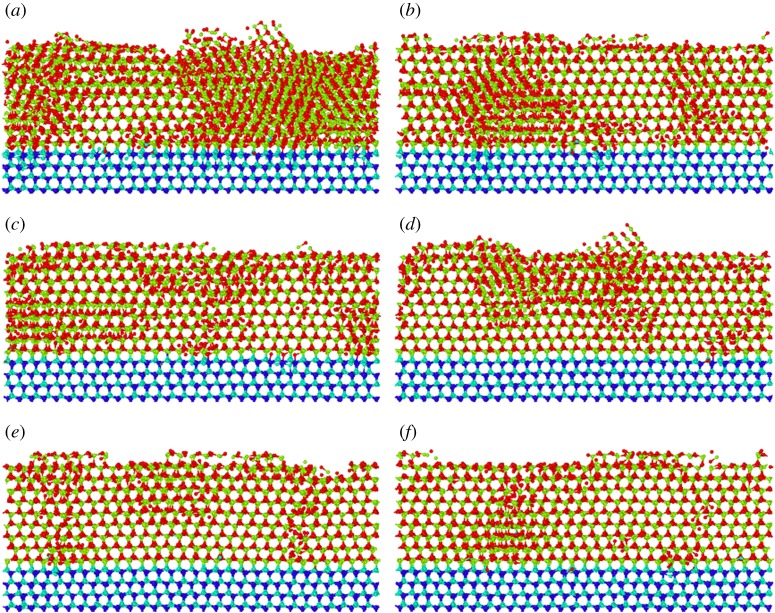
Atomic-scale structure of deposited films under different temperatures with the N : Al flux ratio of 2.0: (*a*) *T* = 1000 K, (*b*) *T* = 1200 K, (*c*) *T* = 1400 K, (*d*) *T* = 1600 K, (*e*) *T* = 1800 K, (*f*) *T* = 2000 K.

**Figure 4.**
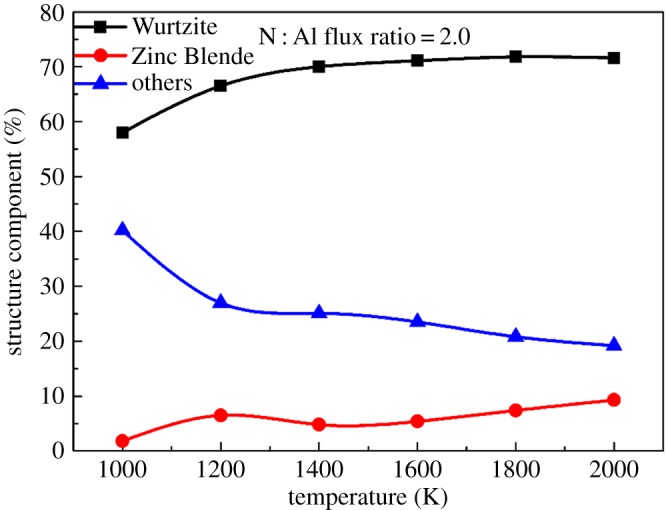
Structure component of the AlN film under different temperatures with an N : Al flux ratio of 2.0.

To explore the lattice arrangement and defects, the atomic-scale structures of deposited AlN films under different growth temperatures are obtained as shown in figure 3. The light blue atoms indicate the N atoms in the substrate, dark blue atoms indicate the Al atoms in substrate, red atoms indicate the injected N atoms and green atoms indicate the injected Al atoms. For a perfect lattice, a hexagonal mesh structure can be observed when seeing along the *x*-axis, as the substrate atoms show. The atomic-scale structure of the deposited AlN film at low temperature (1000 K) contains a high population of defects and even some amorphousness, as shown in figure 3*a*. As the temperature increases from 1000 K to 1800 K, the deposited films exhibit much better developed crystalline features. The deposited AlN film shows regular epitaxial planes and contains a low defect concentration at 1800 K, as shown in figure 3*e*. The crystalline feature gets saturated from 1800 K to 2000 K. To quantitatively compare the atomic structure of deposited films under different temperatures, figure 4 shows the structure component of AlN films with a N : Al flux ratio of 2.0. Three types of structures: Wurtzite, Zinc Blende and other structure are counted. Among them, Wurtzite is the ideal structure of the AlN film. Figure 4 shows an increased component of Wurtzite as the growth temperature increases from 1000 K to 1800 K, which indicates the improved crystallinity as the temperature increases from 1000 K to 1800 K. It is obvious that high temperature is good for getting better crystallinity. With the diffusion barrier of 1.17 eV, it is difficult for Al adatoms to diffuse and adsorb [22,34]. Increasing the temperature increases the mobility of Al adatoms, promoting better crystallinity of deposited films. The effect of temperature on crystalline quality has been experimentally explored. Claudel *et al.* [36] found that the crystalline quality of epitaxial AlN layers grown on AlN templates increased with increasing deposition temperature from 1400°C to 1500°C, which is clearly consistent with our simulation.

**Figure 5.**
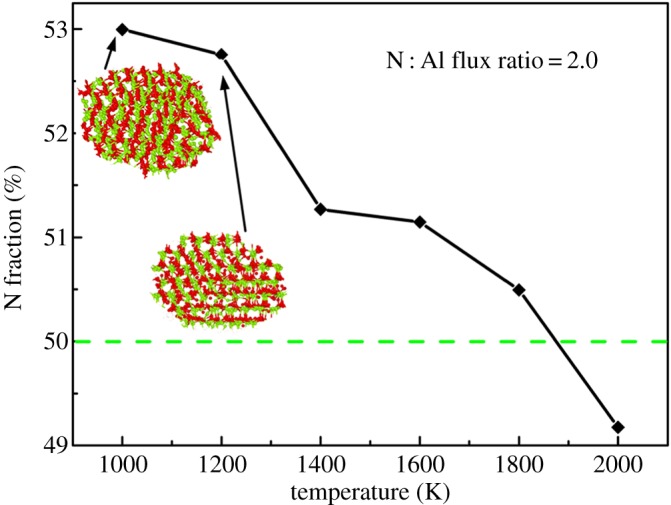
N fraction of deposited films with an N : Al flux ratio of 2.0 under different deposition temperatures ranging from 1000 K to 2000 K, with an increment of 200 K.

The ideal stoichiometry of the AlN film is 1, which means the perfect N fraction in AlN is 50%. Greater than or less than 50% means excess of N or Al atoms, correspondingly, both of which cause defects in deposited AlN film. Here, the N fractions of deposited AlN films with an N : Al flux ratio of 2.0 under different temperatures are calculated in figure 5. It is clear that as the temperature increases, the N fraction decreases. An N fraction much greater than 50% appears at 1000 K and 1200 K; the related defect clusters are shown as insets in figure 5. It is approximately 50% from 1800 K to 2000 K. This indicates that a higher N : Al flux ratio is needed to get an ideal stoichiometric ratio as the temperature increases. Combining figures 3 and 4, we can clearly see that the AlN film at 1800 K has both a relatively good crystallinity and N fraction, which indicates the connection between good crystallinity and a near 50% N fraction.

### 3.2. Effect of N : Al flux ratio

**Figure 6.**
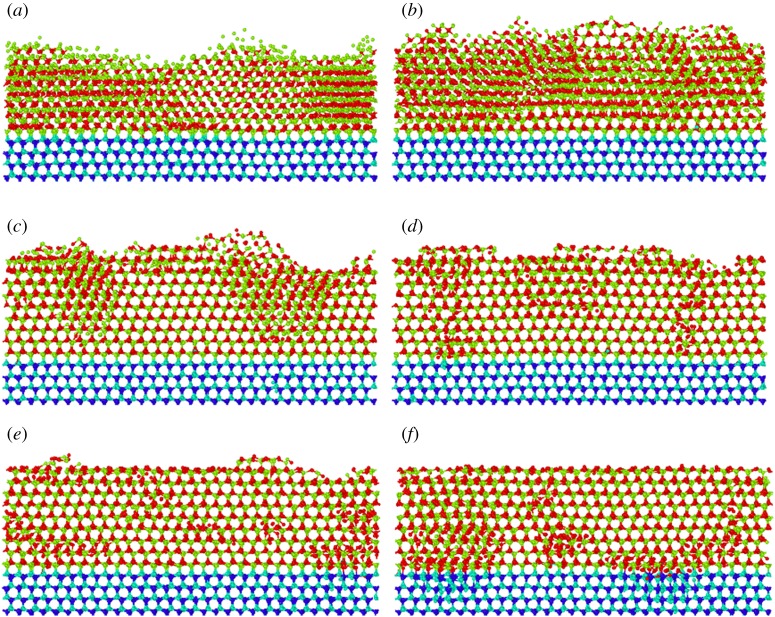
Atomic-scale structure of deposited films at 1800 K with different N : Al flux ratios: (*a*) N : Al flux ratio = 0.8, (*b*) N : Al flux ratio = 1.2, (*c*) N : Al flux ratio = 1.6, (*d*) N : Al flux ratio = 2.0, (*e*) N : Al flux ratio = 2.4 and (*f*) N : Al flux ratio = 2.8.

**Figure 7.**
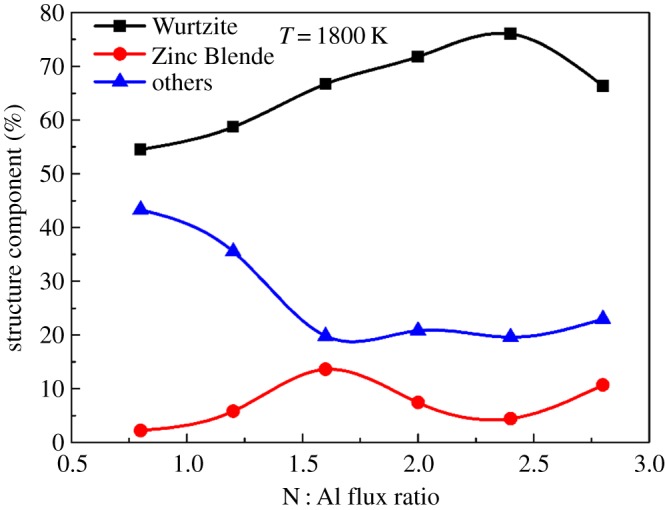
Structure component of the AlN film under different temperatures with an N : Al flux ratio of 2.0.

As we know from the above, the N : Al flux ratio is an important factor influencing crystallinity as well as stoichiometry. Here, the N : Al flux ratio of deposited AlN at 1800 K is further optimized. The atomic-scale structures of the deposited AlN films at 1800 K are shown in figure 6*a*–*f* with six different N : Al ratios (0.8,1.2, 1.6, 2.0, 2.4 and 2.8). It can be seen in figure 6*a,b* that at a low N : Al flux ratio (0.8 and 1.2), the atoms are arranged in great disorder. As the N : Al flux ratio increases to 1.6, there is still some disorder but the overall film crystallinity is improved. When the N : Al flux ratio is further increased to 2.0 and 2.4, the crystallinity of the film is further improved. Little disorder and few defects remain. At the N : Al flux ratio of 2.8, the film shows a little degradation of crystallinity, as in figure 6*f*. Epitaxial film growth is clearly promoted by increasing the N : Al ratio from 0.8 to 2.4. Figure 7 shows the structure component of AlN films at 1800 K under different N : Al flux ratios. The component of Wurtzite increases as the N : Al flux ratio increases from 0.8 to 2.4 and decreases as the N : Al flux ratio increases from 2.4 to 2.8, indicating the improved crystallinity as the N : Al flux ratio increases from 0.8 to 2.4 and degraded crystallinity as the N : Al flux ratio increases from 2.4 to 2.8. Obviously, a N : Al flux ratio of 2.4 is the optimal flux ratio for the AlN film grown at 1800 K.

It should be pointed out that the experimentally applied V/III flux ratio [37,38] is not equal to the N : Al flux ratio in our simulation because of too many pre-reactions between the Al source and the N source [39]. But using the beam equivalent pressure ratio to represent the N : Al flux ratio can approximately approach our simulation conditions. The N plasma that dominates the reaction could be observed. Kaneko *et al*. [40] found that the emission spectrum of N plasma is dominated by a series of sharp atomic N emission peaks (N*) during the growth of AlN. Thus, the N : Al flux ratio could be denoted by N* : Al. They found that the high Al : N ratios (1.67 and 1.18) leads to bad AlN crystal quality, which indicates that a lower Al : N ratio (lower than 1.18), in other words, a higher N : Al ratio (higher than (1/1.18) = 0.85) is needed to get good crystal quality. Meanwhile, our simulation also indicates that an N : Al flux ratio higher than 1 is needed to get good crystallinity under different temperatures. The trends in the experiment and our simulation are consistent.

**Figure 8.**
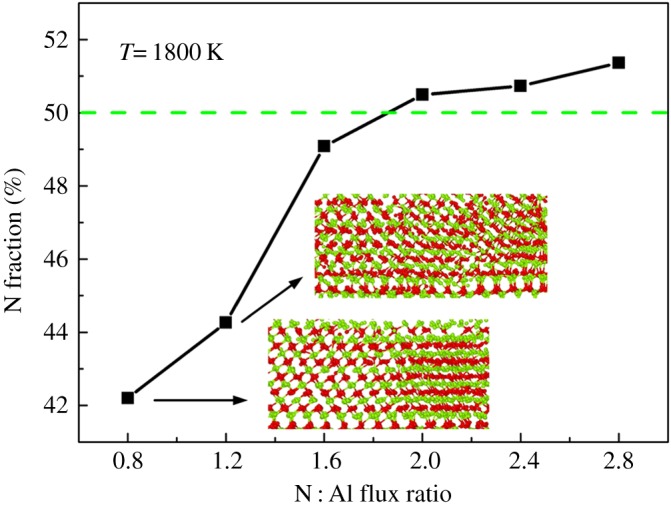
N fraction of deposited films at 1800 K with different N : Al flux ratios from 0.8 to 2.8, with an increment of 0.4.

The N fraction of deposited AlN at 1800 K is explored as a function of N : Al flux ratio in figure 8. The deposited film has a N fraction below 50% when the N : Al flux ratio increases from 0.8 to 1.6 and becomes approximately 50% at the N : Al flux ratios of 2.0 and 2.4. Sub-stoichiometric surfaces are formed when the N : Al flux ratio is below 2.0. As the N : Al flux ratio increases to 2.8, the N fraction of the deposited film becomes greater than 50%. Less or greater than 50%, both cause defects and degraded crystallinity. The related defects cluster at the N : Al flux ratios of 0.8 and 1.2 are shown as insets in figure 8. This could be definitely proved by figures 6 and 7.

**Figure 9.**
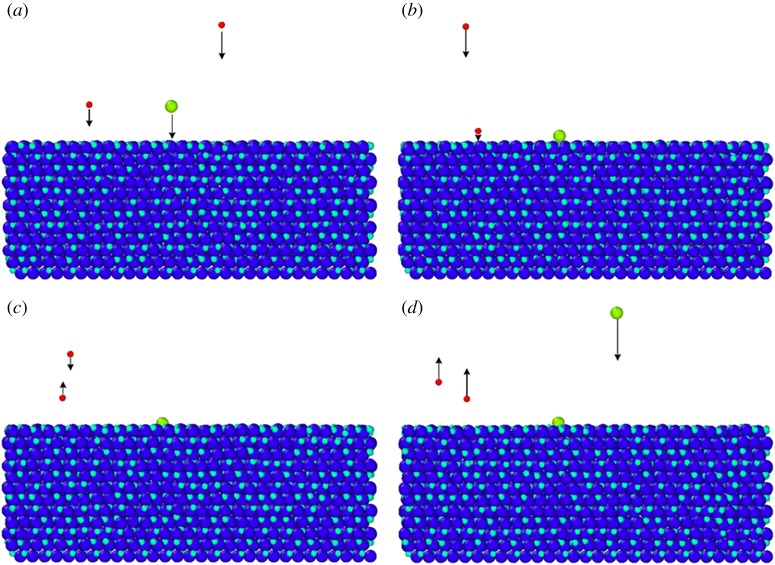
Time-resolved process for N and Al atoms interacting with substrate atoms at 1800 K with an N : Al flux ratio of 2.0. (*a*) *t* = 111 ps, (*b*) *t* = 112 ps, (*c*) *t* = 113 ps (*d*) *t* = 114 ps (the light blue atoms indicate the N atoms in the substrate, dark blue atoms indicate the Al atoms in substrate, red atoms indicate the injected N atoms and green atoms indicate the injected Al atoms).

Figures 5 and 8 show a phenomenon that the N fraction decreases as the temperature increases and increases as the N : Al flux ratio increases. The ideal N : Al flux ratio at 1800 K is 2.4 (greater than 1). Here, time-resolved images of the interaction between injected Al and N atoms with substrate atoms on the surface are shown in figure 9*a*–*d*. There are two N atoms and one Al atom moving towards the substrate in figure 9*a*, then one N and one Al atom impact with atoms on the substrate surface correspondingly in figure 9*b*. In figure 9*c*, the impacting Al atom is bonded to the atoms on the substrate surface, while the impacting N atom rebounds and moves away. Figure 9*d* shows another rebounded N atom and a new impacting Al atom. The different mechanism of Al and N atoms interacting with substrate atoms means that the number of injected N atoms is always more than the number of injected Al atoms and the number of injected N atoms is always greater than deposited N atoms. Film with good crystallinity requires a near stoichiometry. Thus, the AlN film is always grown with a N : Al ratio greater than 1.

The crystal quality of the film is closely related to the stress in it. So the stress of the films is discussed. Here we discuss the average normal stress and the average mean biaxial stress of the films at 1800 K with the N : Al flux ratios of 2.0, 2.4 and 2.8. The deposited films are divided into 26 layers along the *z*-axis while ignoring the fixed atoms at the bottom of the substrate. The height of each layer is 2 Å. Then the average normal stress and the average mean biaxial stress of each layer are calculated by formulae (2.1) and (2.2), respectively.

**Figure 10.**
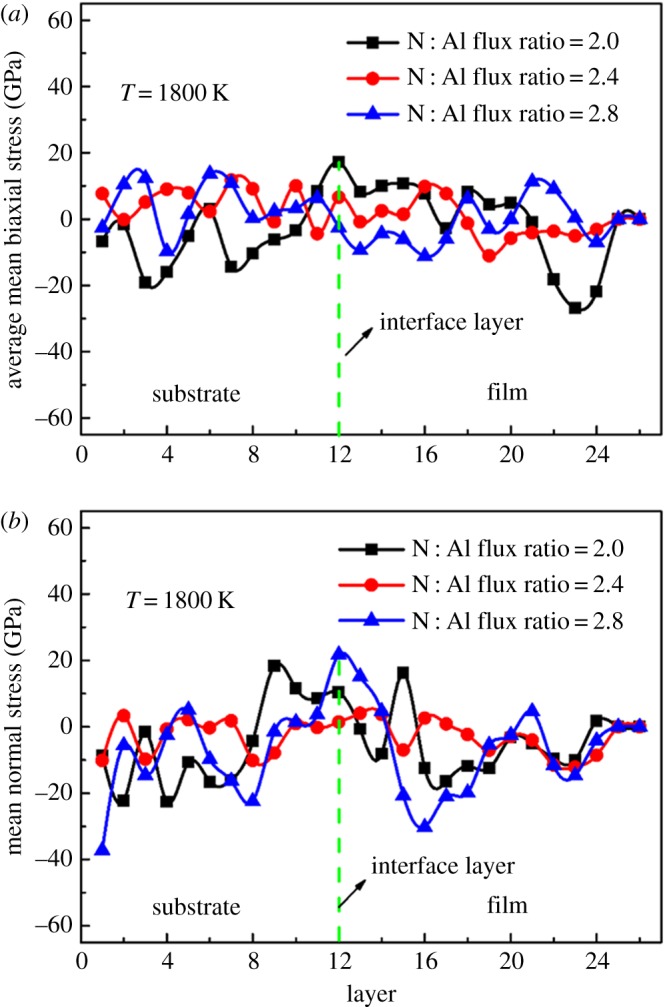
The average mean biaxial stress (*a*) and the average normal stress (*b*) of the films after deposition at 1800 K with different N : Al flux ratios (2.0, 2.4 and 2.8).

From figure 10*a*, we can see that the fluctuation of the average mean biaxial stress at an N : Al flux ratio of 2.4 is minimal and maximal at an N : Al flux ratio of 2.0. Furthermore, the biggest fluctuation range at the N : Al flux ratio of 2.0 appears at the layers where the majority of defects appears, as shown in figure 6*d*. Similarly, when comparing figures 10*b* and 6, we find that the fluctuation of the average normal stress is minimal at the N : Al flux ratio of 2.4 and maximal at the N : Al flux ratio of 2.8. Moreover, the biggest fluctuation range at the N : Al flux ratio of 2.8 appears at the layers where the majority of defects appears, as shown in figure 6*f*. Taken together, the defects and stresses are interrelated. The defects appear where stresses occur. Furthermore, good crystallinity at 1800 K is obtained when the fluctuation of both the average mean biaxial stress and the average normal stress are minimal.

## 4. Conclusion

We study the atomic growth mechanism of AlN grown on c-plane AlN by MD simulations. The effects of temperature and N : Al flux ratio are investigated in detail. We find that as the temperature increases from 1000 K to 2000 K at an N : Al flux ratio of 2.0, the growth rate decreases. Meanwhile, the crystallinity of deposited AlN is distinctly improved as the temperature is increased from 1000 K to 1800 K and becomes saturated between 1800 K and 2000 K. Increasing growth temperature effectively reduces amorphousness and defects, which results in good crystallinity. The crystallinity of the deposited film at 1800 K also increases when increasing the N : Al flux ratio from 0.8 to 2.4 and degrades a little at an N : Al flux ratio of 2.8. The N fraction of the film under different temperatures and N : Al flux ratios are also studied. The results indicate that an N : Al flux ratio above 1 is applied to all simulations for the preferential evaporation of injected N atoms on N-terminated AlN substrate surface. Stoichiometry is closely related to crystallinity of deposited films. Film with good crystallinity is connected with a near 50% N fraction. The average normal stress and the average mean biaxial stress of the deposited film at 1800 K with N : Al flux ratios of 2.0, 2.4 and 2.8 are also calculated, and the results show that good crystallinity is obtained when fluctuation of stresses are minimum and the defects appear where stresses occur.

## Supplementary Material

Model of AlN substrate;Tersoff potential;Cfg files of finally deposited AlN film

## Data accessibility

The datasets supporting this article have been uploaded as part of the electronic supplementary material.
